# Cancer immunotherapies targeting the PD-1 signaling pathway

**DOI:** 10.1186/s12929-017-0329-9

**Published:** 2017-04-04

**Authors:** Yoshiko Iwai, Junzo Hamanishi, Kenji Chamoto, Tasuku Honjo

**Affiliations:** 1 Department of Molecular Biology, School of Medicine, University of Occupational and Environmental Health Japan, Kitakyushu-shi, Fukuoka 807-8555 Japan; 2 Department of Immunology and Genomic Medicine, Graduate School of Medicine, Kyoto University, Yoshida Konoe-cho, Sakyo-ku, Kyoto, 606-8501 Japan; 3 Department of Gynecology and Obstetrics, Graduate School of Medicine, Kyoto University, 54 Kawahara-cho, Shogoin, Sakyo-ku, Kyoto, 606-8507 Japan

**Keywords:** PD-1, PD-L1, Cancer immunotherapy, Immune checkpoint

## Abstract

Immunotherapy has recently emerged as the fourth pillar of cancer treatment, joining surgery, radiation, and chemotherapy. While early immunotherapies focused on accelerating T-cell activity, current immune-checkpoint inhibitors take the brakes off the anti-tumor immune responses. Successful clinical trials with PD-1 monoclonal antibodies and other immune-checkpoint inhibitors have opened new avenues in cancer immunology. However, the failure of a large subset of cancer patients to respond to these new immunotherapies has led to intensified research on combination therapies and predictive biomarkers. Here we summarize the development of PD-1-blockade immunotherapy and current issues in its clinical use.

## Background

Cancer immunotherapy, although controversial for many years, reached a turning point in 2014. Antibodies that specifically block PD-1 were approved for melanoma in 2014 and for non-small-cell lung cancer (NSCLC) in 2015 in the United States, European Union, and Japan. The success of clinical trials with novel drugs targeting immune-checkpoint molecules such as PD-1 led to a paradigm shift in cancer treatment. Since a PD-1 blockade targets lymphocytes rather than cancer cells, it has a long-term therapeutic effect that persists even when cancers cause mutations. Furthermore, the PD-1 blockade is effective against many types of tumors because it enhances the anti-tumor activity of cytotoxic T lymphocytes (CTLs), which recognize various tumor-specific antigens. Several companies are currently conducting phase 3 trials for different tumor types, including renal-cell cancer (RCC), bladder cancer, head and neck cancer, ovarian cancer, and brain cancer. Although PD-1 blockade has dramatically improved the response rate for several cancers, three questions remain to be answered: 1) Why do some patients not respond to PD-1 blockade? 2) What is the best combination therapy using PD-1 blockade? 3) What predictive biomarkers can be used to distinguish responsive and unresponsive patients? Here we review the development of immunotherapy targeting the PD-1/PD-L1 signaling pathway and discuss the issues that still need to be resolved in clinical studies.

## History of cancer immunotherapy

The concept of cancer immunotherapy goes back to the late nineteenth century. In 1891, a young New York surgeon named William Coley began intra-tumoral injections of bacterial products and observed tumor shrinkage in patients with sarcoma [1]. Almost a century later, the role of dendritic cells and their receptors in sensing microorganisms in the innate immune system was discovered [2, 3]. The molecular identification of cancer antigens created new approaches for effective immunotherapies [4]. In addition, the importance of IFN-γ and adaptive immunity in cancer immunosurveillance was demonstrated in preclinical tumor models using IFN-γR^−/−^ and RAG2^−/−^ mice [5]. These findings stimulated research into strategies to induce anti-tumor responses and led to immunotherapies such as cytokine therapy, peptide vaccine, dendritic-cell vaccine, and adoptive T-cell therapy. Most of these therapies were unsuccessful, and one primary reason was a lack of understanding of the existence and importance of immune checkpoints [6].

## Immune checkpoints

**Fig. 1:**
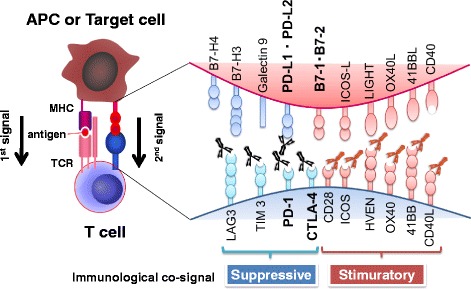
Costimulatory molecules that positively or negatively regulate immune responses

T-cell activating (accelerator) and inhibitory (brake) receptors regulate the balance between immune response and immune tolerance. The activation of naïve T cells requires both antigen presentation (signal 1) and a second signal sent through costimulatory receptors such as CD28 (signal 2) (Fig. 1) [7]. When ligated by B7 molecules such as CD80 (B7-1) or CD86 (B7-2), CD28 coreceptors on T cells deliver a positive costimulatory signal, whereas CTLA-4 coreceptors deliver a negative co-inhibitory signal. PD-1, like CTLA-4, belongs to the CD28 family and delivers a negative signal when it interacts with its ligands, PD-L1 (B7-H1 or CD274) and PD-L2 (B7-DC or CD273), which belong to the B7 family (Fig. 1) [8–10].

T cells have immune checkpoints such as PD-1 and CTLA-4 to reduce autoimmune responses against self-tissues by overly exuberant immune responses to infection. While most cancer immunotherapies accelerate T-cell activity, immune-checkpoint inhibitors release the immune system’s brakes to unleash anti-tumor immune responses.

## Immunoinhibitory mechanism by PD-1

**Fig. 2:**
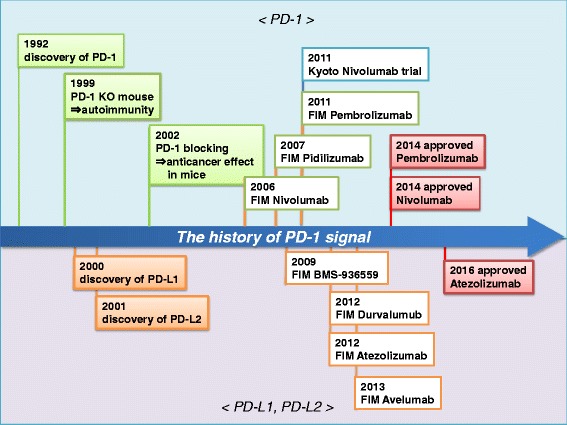
History of PD-1 research. Abbreviations: FIM, first in man; approved, FDA-approved; NCT, “National Clinical Trial” registry number in ClinicalTrials.gov in the United States; FIM Pembrolizumab (P07990/MK-3475-001/KEYNOTE-001), NCT01295827; FIM Pidilizumab (CT-011), NCT00532259; FIM BMS-936559 (MDX-1105), NCT00729664; FIM Atezolizumab, NCT01693562; FIM Durvalumab (MEDI4736), NCT01693562; FIM Avelumab, NCT01772004

PD-1 was discovered in 1992 (Fig. 2). Ishida et al. isolated the gene that encodes PD-1 by cDNA subtraction in apoptosis-induced murine T-cell lines. PD-1 is mainly expressed on activated CD4^+^ T cells and CD8^+^ T cells as well as on B cells in the periphery [11–13]. The activation-induced expression of PD-1 suggests that PD-1 regulates late-phase immune responses (effector phase, memory response, chronic infection, etc.) in the peripheral tissues, rather than the early induction phase in the lymphoid organs.

PD-1’s extracellular region consists of a single IgV-like domain, and its cytoplasmic region contains an immunoreceptor tyrosine-based inhibitory motif (ITIM) and an immunoreceptor tyrosine-based switch motif (ITSM). Upon ligation with its physiological ligand (PD-L1 or PD-L2), PD-1 suppresses T-cell activation by recruiting SHP-2, which dephosphorylates and inactivates Zap 70, a major integrator of T-cell receptor (TCR)-mediated signaling [14, 15]. As a result, PD-1 inhibits the T-cell proliferation and effector functions such as IFN-γ production and cytotoxic activity.

The promoter region of the *Pdcd1* gene has two transcription-factor binding sites that are critical in regulating PD-1 expression. In naïve T cells, TCR-mediated calcium influx initiates *Pdcd1* transcription by activating NFATc1, which binds to the 5′-promoter region of the *Pdcd1* gene (at position −1160 relative to the transcription start site) [16]. On the other hand, in chronically activated (“exhausted”) T cells, interferon-α (IFN-α) causes prolonged *Pdcd1* transcription by the binding of the transcription factor IRF9 to the *Pdcd1* promoter (at position −1040 relative to the transcription start site) [17]. In addition, the *Pdcd1* promoter region (located 500–1500 base pairs upstream of the initiation codon) is demethylated during chronic infection, causing high PD-1 expression in exhausted CD8^+^ T cells [18]. While exhausted CD8^+^ T cells express high eomesodermin (EOMES), which is regulated by transcription factor FoxO1, FoxO1 also binds the *Pdcd1* promoter and enhances PD-1 expression [19].

## PD-1 deficiency and autoimmunity

PD-1’s immunoinhibitory function was elucidated by characterizing the autoimmune phenotype of PD-1–deficient mice, in which PD-1 deficiency leads to a loss of peripheral tolerance and the subsequent development of autoimmunity (Fig. 2) [20, 21]. PD-1–deficient mice develop different autoimmune diseases depending on their genetic background: C57BL/6-Pdcd1^−/−^ mice develop lupus-like arthritis and glomerulonephritis with IgG3 and C3 deposits [20]. BALB/c-Pdcd1^−/−^ mice develop fetal dilated cardiomyopathy with a concomitant production of autoantibodies against cardiac troponin I [21, 22]. NOD-Pdcd1^−/−^ mice develop type I diabetes with extensive destruction of the islets [23]. Furthermore, PD-1–deficient mice crossed with H-2Ld–specific 2C-TCR transgenic mice on the H-2b/d background develop a chronic and systemic graft-versus-host-like disease [20]. These findings indicate that PD-1 negatively regulates immune responses and is essential for maintaining peripheral tolerance.

## Distinct physiological functions of PD-1 and CTLA-4

Although PD-1 and CTLA-4 are both induced on activated T cells, they are expressed at different stages of the immune response. CTLA-4 is closely related to CD28, but binds CD80 and CD86 with a much higher affinity than does CD28 [24]. CTLA-4 is constitutively expressed on regulatory T (Treg) cells, and transiently expressed on activated T cells at the early induction phase after antigen stimulation [25]. In contrast, PD-1 is expressed on activated T cells at the late effector phase, and high and persistent PD-1 expression has been observed on exhausted CD8^+^ T cells during chronic viral infection [26, 27]. CTLA-4 is continuously internalized by interactions with the adaptor complex AP2 and is almost undetectable on the cell surface during T-cell activation; in contrast, PD-1 lacks an AP2-binding motif, which may allow its sustained expression on the surface of activated T cells [28].

Although both PD-1 and CTLA-4 are immune checkpoints, they regulate different phases of the immune response. CTLA-4 blocks early T-cell activation in the lymphoid organs, whereas PD-1 inhibits effector T-cell activity at later-stage immune responses in peripheral tissues and in the tumor microenvironment. PD-1 and CTLA-4 also have distinct inhibitory mechanisms. CTLA-4 completely blocks costimulation by CD28 through its stronger affinity for B7 molecules, whereas PD-1’s inhibitory function depends mostly on its recruitment of SHP-2 [29–32]. These differences in expression and inhibitory mechanisms are probably responsible for the different autoimmune phenotypes of PD-1 and CTLA-4 deficiency. CTLA-4-deficient mice develop devastating autoimmune diseases and massive and systemic lymphoproliferation, and die within 5 weeks of birth [33]. In contrast, PD-1–deficient mice remain relatively healthy into later stages of life, eventually developing relatively mild, organ-specific autoimmune symptoms depending on their genetic background [20, 21]. Consistent with the phenotypes of PD-1–knockout and CTLA-4–knockout mice, PD-1 inhibitors are less toxic than CTLA-4 inhibitors [34, 35].

## Identification of PD-1 ligands

PD-L1 and PD-L2 were identified as PD-1 ligands in 2000 and 2001, respectively (Fig. 2) [9, 10]. PD-L1 and PD-L2 are type I transmembrane proteins with IgV- and IgC-like domains in the extracellular region. PD-L1 is broadly expressed in both lymphoid and non-lymphoid tissues. PD-L1 is upregulated upon activation on hematopoietic cells, especially on antigen-presenting cells (APCs) such as dendritic cells, macrophages/monocytes, and B cells [36, 37]. PD-L1 is also expressed on activated T cells. Importantly, PD-L1 is expressed on non-lymphoid cells, including parenchymal cells and vascular endothelial cells in the peripheral tissues, and is upregulated by IFN-γ and other inflammatory cytokines secreted by activated T cells [23, 26, 38]. The expression of PD-L1 in peripheral tissues rather than on professional APCs is crucial for preventing autoimmune damage to tissues [39]. Interestingly, PD-L1 is expressed in various tumor cells and virus-infected cells. The expression of PD-L1 on target cells allows PD-1 to directly inhibit T-cell effector functions against the target cell. Unlike PD-L1, which is expressed in many different tissues, PD-L2 is expressed only on APCs such as dendritic cells and macrophages [37].

## Regulation of tumor immunity by PD-1

The PD-1/PD-L1 signaling pathway is crucial in dampening immunosurveillance for tumors. Tumors can escape host immune surveillance by expressing PD-L1, which negatively regulates immune responses by interacting with PD-1 on T cells (Fig. 1) [40]. Indeed, data from clinical samples indicate that the high expression of PD-1 ligands on tumors is correlated with a poor prognosis [41, 42].

The first evidence of the PD-1/PD-L1 pathway’s involvement in tumor immunity was found in animal models [40]. PD-L1 overexpression on P815 mastocytomas was shown to inhibit the cytolytic activity of CD8^+^ T cells by engaging PD-1 in vitro, and to markedly enhance tumorigenesis and tumor invasiveness in vivo. Anti–PD-L1 treatment inhibited the growth of PD-L1–expressing P815 tumor cells, and of J558L myeloma cells, which endogenously express PD-L1. Importantly, no tumors developed in *Pdcd1* ^−/−^ mice after their inoculation with J558L cells. These results revealed the effectiveness of the PD-1/PD-L1 blockade for tumor therapy.

Although a CTLA-4 blockade enhances immune responses against immunogenic tumors such as lymphoma in animal models, it is not effective as a single agent against poorly immunogenic tumors such as B16 melanoma [43–45]. However, even as a single agent, a PD-1 blockade was found to be therapeutic against B16 melanoma in a liver metastasis model [46]. These results suggested that PD-1 blockade can be successfully applied to metastatic tumors, and that it has a stronger therapeutic potential than does CTLA-4 blockade.

## Clinical application of the PD-1/PD-L1 blockade

Several clinical studies have reported that PD-L1 overexpression is related to a poor prognosis for several types of tumors, including renal-cell carcinoma, bladder cancer, esophageal cancer, pancreatic cancer, gastric cancer, hepatocellular carcinoma, and ovarian cancer [41, 47–53]. In ovarian cancer, PD-L1 expression is negatively correlated with the number of intra-epithelial infiltrating CD8^+^ T cells, suggesting that the PD-L1 expression on tumor cells prevents CD8^+^ T cells from infiltrating tumor sites [50]. These studies indicated that blocking PD-1 signaling might improve clinical outcomes for patients with these malignancies. In 2006, a proof-of-concept clinical study using a PD-1 signal inhibitor against treatment-resistant solid tumors was initiated in the United States (Fig. 2) [54].

A fully humanized monoclonal antibody (mAb) against PD-1 (nivolumab; also known as ONO4538, MDX-1106, or BMS-936558) was first developed using genetically modified mice carrying loci encoding human immunoglobulins. The IgG4 isotype of nivolumab minimizes complement activity or antibody-dependent cell-mediated cytotoxicity (ADCC) [54]. This antibody carries a serine-to-proline substitution at position 228 to minimize the effect of ADCC against activated T cells. Clinical trials of nivolumab began in 2006 in the United States and in 2009 in Japan (Fig. 2). The phase 1 study of nivolumab showed cumulative response rates of 18% for NSCLC, 28% for melanoma, and 27% for renal carcinoma. Grade 3 or 4 drug-related adverse events occurred in 14% of the patients [35]. Notably, nivolumab has demonstrated durable clinical activity as a single agent, with far fewer side effects than are seen with ipilimumab, a mAb against CTLA-4 [34, 35]. A clinical trial using anti–PDL1 mAbs (BMS-936559 or MDX-1105) showed relatively low response rates compared to an anti–PD-1 mAb [55].

The PD-1 blockade approach has unique features compared to standard therapies. Conventional chemotherapies usually target a particular molecule in the tumor cells. The tumor cells can escape the therapy with mutations of the target molecules, leading to rapid regression. However, a PD-1 blockade is applicable to a wide range of cancers and provides a response over a longer period because it activates an anti-tumor immune system that can target mutated proteins [56]. In addition, PD-1 blockade has a significantly lower rate of high-grade toxicities than other immunotherapies or standard therapies, because the anti-tumor immunity preferentially recognizes tumor-derived antigens, not self-antigens. In a phase 3 study comparing nivolumab to the plant alkaloid chemotherapy drug docetaxel in 272 patients with advanced squamous-cell NSCLC, the response rate was 20% with nivolumab versus 9% with docetaxel [57]. The overall survival rate at 1 year was 42% with nivolumab versus 24% with docetaxel. The frequency of grade 3 or 4 treatment-related adverse events was much lower in the nivolumab group (7%) than in the docetaxel group (55%).

To date, at least 500 clinical studies with PD-1 signal inhibitors have been conducted with nine types of antibodies from eight pharmaceutical companies (Table 1 and Fig. 2) on at least 20 types of solid and hematological malignant tumors (Table 2) [58]. The total number of subjects worldwide is more than 20,000, according to a clinical trials database managed by the U.S. National Institutes of Health (https://clinicaltrials.gov/[CTG]). The U.S. Food and Drug Administration (FDA) approved nivolumab for patients with unresectable or metastatic melanoma in 2014, for NSCLC in 2015, and for classical Hodgkin’s lymphoma and RCC in 2016. The FDA also approved pembrolizumab for melanoma in 2014 and for NSCLC in 2015. Atezolizumab, an anti–PD-L1 antibody, was approved for unresectable bladder cancer and for NSCLC in 2016.

**Table 1:** PD-1 signal inhibitors (anti–PD-1 and anti–PD-L1 antibodies) in clinical trials

Target	Agent	IgG class	Company	Approved
PD-1	nivolumab (Opdivo®, BMS-936558, MDX1106)	Human IgG4	Bristol-Meyers Squibb/Ono	Melanoma^U, E, J^ Lung cancer^U, E, J^ Kidney cancer^U, J^ Hodgkin’s lymphoma ^U, E, J^ Head and neck cancer ^U^ Urothelial cancer ^U^
	pembrolizumab (Keytruda®MK-3475, lambrolizumab)	Humanized IgG4	Merck	Melanoma^U, E, J^ Lung cancer^U, E, J^ Head and neck cancer^U^
	pidilizumab (CT-011)	Humanized IgG1k	Cure Tech	
	AMP-224	PD-L2 IgG2a fusion protein	Amplimmune/GlaxoSmith Klein	
	AMP-514 (MEDI0680)	PD-L2 fusion protein	Amplimmune/GlaxoSmith Klein	
	PDR001	Humanized IgG4	Novartis Pharmaceuticals	
PD-L1	BMS-936559 (MDX1105)	Human IgG4	Bristol-Meyers Squibb	
	atezolizumab (Tecentriq®, MPDL3280A)	Humanized IgG1k	Roche/Genentech	Urothelial cancer^U^ Lung cancer^U^
	durvalumab (MEDI4736)	Human IgG1k	MedImmune/AstraZeneca	
	avelumab (MSB0010718C)	Human IgG1	Merck Serono/Pfizer	

All antibodies used in clinical trials as of September 1, 2016 were extracted from ClinicalTrials.gov

*Abbreviations*: *U* U.S. Food and Drug Administration (*FDA*) approved; *E* European Medicines Agency (*EMA*) approved, *J* Japanese Pharmaceutical and Medical Devices Agency (*PMDA*) approved

**Table 2:** Clinical effects of monotherapeutic PD-1 signal inhibitors on several types of malignancies

Target	Agent	Phase	Clinical effect	Reference
melanoma	pembrolizumab	2	6MOS 34% (2 mg/kg) vs. 38% (10 mg/kg), vs 16% :docetaxel (*n* = 540)	[88]
		3	1 year-OS 74% (2wks) vs. 38% (3wks), vs 11% :docetaxel (*n* = 834)	[89]
	nivolumab	3	1 year-OS 73% vs 42% (dacarbazine) (*n* = 418)	[89]
		3	ORR 32% vs. 11% (dacarbazine) (*n* = 405)	[90]
non-small cell lung cancer	pembrolizumab	1	ORR 19.4%, mOS12.5 M (total), ORR 45.2% (*n* = 72, PD-L1+) (*n* = 495)	[91]
	nivolumab	3	mOS 9.2 M (vs 6.0 M:docetaxel) (*n* = 272)	[57]
		3	mOS12.2 M (vs 9.7 M:docetaxel (*n* = 582)	[92]
	durvalumab	1/2	ORR 14% (*n* = 149, total), 23% (PD-L1+)	[72]
	atezolizumab	2	ORR 15% (*n* = 144, total), 38% (*n* = 24, PD-L1+)	[93]
small cell lung cancer	nivolumab	1/2	ORR 18% (*n* = 40, nivo), 17% (*n* = 46, combined with chemotherapy)	[94]
	pembrolizumab	1	ORR 25% (*n* = 16)	[95]
head and neck cancer	durvalumab	1/2	ORR 12% (*n* = 62)	[96]
	pembrolizumab	1	ORR 24.8% (*n* = 117)	[97]
renal cell cancer	nivolumab	3	ORR 25%, mOS 25.0 M, (vs. ORR 5%, mOS 19Ms in everolimus) (*n* = 821)	[98]
bladder cancer	atezolizumab	1	ORR 26% (*n* = 310, total), 43% (PD-L1+)	[99]
	pembrolizumab	1	ORR 25% (*n* = 33, total), 38% (PD-L1+)	[100]
ovarian cancer	nivolumab	2	ORR 15% (*n* = 20, total), mOS 20.0 M ORR 20% (*n* = 10, 3 mg/kg)	[60]
	avelumab	1	ORR 10% (*n* = 124)	[101]
	pembrolizumab	1	ORR 11.5% (PD-L1+) (*n* = 49)	[63]
uterine endometrial cancer	pembrolizumab	1	ORR 12.5% (PD-L1+) (*n* = 24)	[102]
uterine cervical cancer	pembrolizumab	1	ORR 12.5% (PD-L1+) (*n* = 24)	[103]
uterine sarcoma	nivolumab	1	ORR 0% (*n* = 12)	[104]
gastric cancer	pembrolizumab	1	ORR 31% (*n* = 39)	[79]
esophageal cancer	pembrolizumab	1	ORR 30% (PD-L1+) (*n* = 23)	[105]
DNA mismatch repair deficient colon	pembrolizumab	2	ORR 40% (*n* = 10, MMRd colon), vs 0% (*n* = 18) in MMRw, vs71% (*n* = 7), MMR-non-colon [cholangiocarcinoma, endometrial cancer and pancreatic cancer].)	[73].
DNA mismatch repair deficient endometrial cancer	pembrolizumab	2	ir-ORR 67% (*n* = 9)	[106]
hepatocellular carcinoma	nivolumab	1/2	ORR 9% (*n* = 91), 6 month-OS 69%.	[107]
breast cancer	atezolizumab	1	ORR 12% (*n* = 27)	[108]
	pembrolizumab	1	ORR 19% (*n* = 25) (PD-L1+)	[109]
Merkel cell carcinoma	pembrolizumab	2	ORR 56% (*n* = 25), 6 M-PFS 67%	[110]
thyroid cancer	pembrolizumab	1	ORR 9.1% (*n* = 22), mOS not reached, 1 year-OS 89.9%.	[111]
Hodgikin lymphoma	nivolumab	1	ORR 87%, 24wks-PFS 86%(*n* = 23)	[112]
	pembrolizumab	1	ORR 64% (*n* = 31), 52wks-PFS 46%.	[113]
follicular lymphoma	nivolumab	1	ORR 40% (*n* = 10)	[114]
diffuse large B-cell lymphoma	nivolumab	1	ORR 36% (*n* = 11)	[114]
mycosisfungoides	nivolumab	1	ORR 15% (*n* = 13)	[114]
peripheral T-cell lymphoma	nivolumab	1	ORR 40% (*n* = 5)	[114]

Partially modified from reference [58]. *Abbreviations*: *M* month, *wk* week, *ORR* objective response rate, *OS* overall survival, *PFS* progression-free survival, *irRC* immune-related response criteria, *ASCO* Annual meeting of the American Society of Clinical Oncology, *SGO* Annual meeting of the Society of Gynecologic Oncology, *Abst* Abstract, *MMRd* DNA mismatch repair deficient, *MMRw* DNA mismatch repair wild

Regarding clinical trials for ovarian cancer, we first conducted the principal investigator-initiated two-cohort (1 or 3 mg/kg, *n* = 10 each) phase 2 clinical trial of nivolumab in 20 patients with platinum-resistant recurrent ovarian cancer at Kyoto University Hospital in 2011 (Fig. 2) (UMIN000005714) [59, 60]. The objective response rate at 3 mg/kg was 20%; this included two cases of complete response (CR). For all 20 patients, the response rate was 15% and the durable CR (DCR) was 45%. The median progression-free survival (PFS) and overall survival (OS) were 3.5 and 20.0 months, respectively [60]. In our ongoing follow-up study of this trial, a durable anti-tumor response with nivolumab has been observed in two patients with a complete response for over 1 year. After completing the 1-year nivolumab treatment, these two patients survived without any anti-tumor treatment for over 2 years [61, 62]. Based on this positive result, we are conducting a large randomized phase 2 trial with nivolumab versus standard 2nd-line chemotherapy (*NINJA study*, JapicCTI-153004). So far, at least 30 clinical trials have been completed or are underway for ovarian cancers using the monotherapeutic anti–PD-1 antibody pembrolizumab (response rate [RR] 10%, *n* = 104) [63], the anti–PD-L1 antibody avelumab (RR 10%, *n* = 104) [64], or combinations of these agents with conventional cancer therapies (CTG).

## Combination therapy with blockade of PD-1/PD-L1 signal and new co-signals

Patients who respond poorly or are unresponsive to immunotherapies appear to lack preexisting anti-tumor T-cell responses. One possible approach to overcoming this issue is to combine the two immune-checkpoint inhibitors anti–PD-1 and anti–CTLA-4 (Fig. 1). In a phase 1 study on patients with advanced melanoma, concurrent therapy with nivolumab and ipilimumab induced rapid and durable responses, resulting in an unprecedented 2-year survival rate of over 80%; 53% of the patients had an objective response with more than 80% tumor reduction [65]. However, grade 3 or 4 therapy-related adverse events occurred in 53% of the patients. In this double-blind study, 142 patients with metastatic melanoma randomly received ipilimumab combined with nivolumab or placebo once every 3 weeks for four doses. The objective response rate was significantly higher for patients who received the combined ipilimumab and nivolumab regimen (60%) compared to those treated with ipilimumab monotherapy (11%). The median PFS was 8.9 months with the combination therapy and 4.7 months with ipilimumab alone [66]. Based on this confirmatory trial, the FDA approved this combined nivolumab and ipilimumab therapy for unresectable or metastatic melanoma in 2015. Combined nivolumab and ipilimumab therapy is now being clinically applied to other cancer types, including RCC [67], NSCLC [68], and ovarian cancer (NCT02498600). However, the frequency of grade 3 or 4 immune-related adverse events (irAEs) is over 50%, and this issue remains to be resolved [69].

Several clinical trials are underway for PD-1 inhibitors in combination with other immune-checkpoint inhibitors, immune activators, and chemotherapies (Table 3 and Fig. 1). However, combining immunotherapies with chemotherapies can increase irAEs. For example, compared to PD-L1 mAb (durvalumab) or EGFR inhibitor (osimertinib) monotherapies, combining the two therapies induced a significantly higher risk of interstitial lung disease (2% [*n* = 23 of 1149], 2.8% [35 of 1207], and 38% [*n* = 13 of 34], respectively) [70]. At present, at least 20 clinical trials with combined PD-1 inhibitors and focal radiation therapy and more than five trials combining anti-PD-1 mAb with chemoradiation therapy are underway (CTG).

**Table 3:** Clinical trials of combination therapies with molecularly targeted drugs

PD-1/PD-L1mAb	Combination	Tumor	Reference
PD-1 mAb (Nivolumab)	LAG3 (BMS-986016)	Solid Tumors	NCT01968109
PD-1 mAb (Nivolumab)	B7-H3 (Enoblituzumab)	Solid Tumors	NCT02817633
PD-1 mAb (Pembrolizumab)	B7-H3 (Enoblituzumab)	Solid Tumors	NCT02475213
PD-1 mAb (Nivolumab)	KIR (Lirilumab)	Solid Tumors	NCT01714739
PD-L1 mAb (MEDI4736)	OX40 (MEDI6383)	Solid Tumors	NCT02221960
PD-1 mAb (Nivolumab)	4-1BB (Urelumab)	Solid tumors and B-cell non-Hodgkin lymphoma	NCT02253992
PD-1 mAb (Nivolumab)	ICOS (JTX-2011)	Solid Tumors	NCT02904226
Pd-1 mAb (PDR001)	GITR (GWN323)	Solid Tumors and Lymphomas	NCT02740270
PD-1 mAb (Nivolumab)	CD27 (Varlilumab)	Solid Tumors	NCT02335918
PD-L1 mAb (Atezolizumab)	CD27 (Varlilumab)	Solid Tumors	NCT02543645
PD-1 mAb (Nivolumab)	GM.CD40L (vaccine for NSCLC)	Lung (NSCLC)	NCT02466568
PD-L1 mAb (Atezolizumab)	VEGF inhibitors (Bevacizumab cediranib)	Ovarian Cancer	NCT02659384
PD-L1 mAb (MEDI4736)	PARP inhibitors (Olaparib)	S tumors	NCT02484404
PD-L1 mAb (MEDI4736)	Multi-kinase inhibitor (Sunitinib)	Solid tumors	NCT02484404
PD-1 mAb (Pembrolizumab) with SBRT	Multi-kinase inhibitor (Sunitinib)	TKI refractory mRCC^a^	NCT02599779
PD-L1 mAb (Durvalumab)	EGFR inhibitor (Osimertinib)	Lung (NSCLC)	reference [70]

^a^ Tyrosine kinase inhibitor refractory metastatic recal cell cancer

## Biomarkers for predicting the efficacy of the PD-1-blockade cancer immunotherapy

**Fig. 3:**
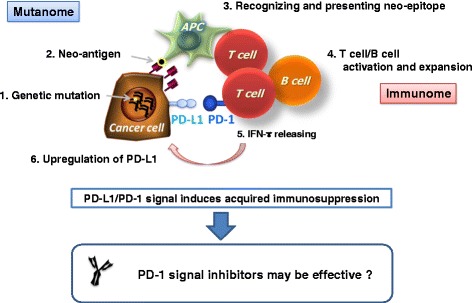
Genomic mutations and PD-1 signal inhibitors. (1) Genetic mutations in cancer cells create neo-antigens. (2) Neo-antigens are expressed on the surface of the cancer cells. (3) Recognition of a neo-antigen as a foreign body by an APC induces a T-cell response, and (4) consequently activates T cells and B cells. (5) Activated T cells release IFN-γ. (6) A cancer cell that is exposed to IFN-γ expresses PD-L1, thereby establishing an acquired immune resistance. In this particular tumor microenvironment, PD-1 signal inhibitors appear to be effective; thus, genome-wide mutation analysis (i.e., Mutanome) of cancer cells using next-generation sequencing technology and diversity analysis of the T-cell or B-cell repertoire (i.e., Immunome) are valuable next strategies for identifying predictive biomarkers [75]. APC, antigen-presenting cell

Potential predictive biomarkers for anti-tumor responses with PD-1 inhibitors can be found among both tumor cell-related factors and host immunological factors. Recent reports identified the frequency of genetic mutations derived from microsatellite instability (MSI) with DNA mismatch repair deficiency (MMRd) in cancer cells as a candidate biomarker [56, 71–73]. Many mutated neo-antigens expressed on the surface of cancer cells are recognized by T cells and B cells as foreign antigens, either directly or through the APC system. Cancer cells exposed to IFN-γ released from activated T cells express PD-L1, thereby establishing an acquired immune resistance [74]; in this case, PD-1 signal inhibitors are more likely to be effective. Thus, genome-wide mutation analysis (i.e., Mutanome) of cancer cells using next-generation sequencing technology and diversity analysis of the T-cell or B-cell repertoire (i.e., Immunome) have attracted a lot of attention as strategies for identifying predictive biomarkers (Fig. 3) [75, 76]. Based on this concept, researchers have examined candidate biomarkers such as the PD-L1 level on tumor cells and the frequency of tumor-infiltrating lymphocytes [77, 78], the levels of IFN-γ–related genes in tumor cells [79, 80], the frequency of mutations in tumor cells [56, 71–73], and the diversity of TCRs in tumor antigen–specific T cells [74, 81, 82]. However, these candidates do not always correspond to a high response according to cancer type. For example, clinical trials of PD-1 inhibitors for squamous-cell lung cancer and ovarian cancer showed no correlation between clinical effect and PD-L1 expression on tumor tissues [57, 60, 64]. A recent report by Hugo et al. revealed that high mutational loads and genes related to T-cell checkpoints, such as CD8A/B, PD-L1, LAG3, and IFN-γ, in tumor tissues were not associated with responsiveness in breast cancer patients [83]. Interestingly, the breast cancer susceptibility gene (BRCA) 2 mutation status is associated with responsiveness to PD-1 mAb therapy [83], while no correlation was found between BRCA2 and avelumab’s clinical effect on ovarian cancer [64]. It is urgent to validate current candidates and to discover new biomarkers for clinical response to PD-1 signal inhibitors.

## Toxicities of PD-1/PD-L1 signal blocking

IrAEs associated with PD-1 blockade therapy include interstitial pneumonitis, colitis with gastrointestinal perforation, type 1 diabetes, severe skin reactions, immune thrombocytopenia, neutropenia and sepsis after corticosteroid therapy, encephalopathy and neurological sequelae, Guillain-Barré syndrome, myelitis, myasthenia gravis, myocarditis and cardiac insufficiency, acute adrenal insufficiency, and nephritis [84–87]. Based on several previous clinical trials, guidelines and specific care algorithms have been established for the identification, early intervention, and management of irAEs [86, 87]. While irAEs can develop at any time, most of the immune toxicities of nivolumab occur within the first 4 months [86, 87]. The median time to onset of irAEs tends to differ depending on the type of toxicity, and can be roughly classified as early (<2 months: skin, gastrointestinal, or hepatic irAEs) or late (>2 months: pulmonary, endocrine, and renal-related irAEs). To treat new types of adverse events and to reduce the frequency of immunological toxicities, oncologists should form and collaborate with networks of organ-specific medical doctors, pharmacists, and nurses.

## Conclusion

Basic and translational studies in the 20 years since PD-1’s discovery have demonstrated the concept of immune surveillance in mice and humans. The recovery of T-cell anergy by blocking PD-1 signals on T cells yielded incredible clinical benefits for several types of malignancies. Nevertheless, there is a still a great deal of exploratory research needed to clarify the fundamental mechanism and predictive biomarkers for the efficacy and adverse effects of this therapeutic strategy. To advance the development of PD-1 signal inhibitors in cancer therapy, it is important to continue both translational and reverse-translational research approaches, including molecular and genomic studies to elucidate the interactions between host and tumor cells.
